# Biomechanical Comparison of Three Internal Fixation Techniques for Stabilizing Posterior Pelvic Ring Disruption: A 3D Finite Element Analysis

**DOI:** 10.1111/os.12431

**Published:** 2019-03-21

**Authors:** Pan Hu, Tao Wu, Hui‐zhi Wang, Xin‐zheng Qi, Jie Yao, Xiao‐dong Cheng, Wei Chen, Ying‐ze Zhang

**Affiliations:** ^1^ Department of Orthopaedics Third Hospital of Hebei Medical University Shijiazhuang China; ^2^ International Research Center for Implantable and Interventional Medical Devices, School of Biological Science and Medical Engineering Beihang University Beijing China

**Keywords:** Biomechanics, Implant, Pelvic fracture, Pelvic ring

## Abstract

**Objective:**

To compare the biomechanical stability and compatibility of two iliosacral screws (ISS), a tension band plate (TBP), and a minimally invasive adjustable plate (MIAP) for treating Tile C pelvic fractures.

**Methods:**

Three groups of finite element models of the intact pelvis, including the main ligament and the proximal one‐third of both femurs, were developed to simulate vertical sacral fractures and treated with the three abovementioned internal fixation techniques. A 500 N vertical load, a 500 N vertical load plus a 10 Nm moment of forward sagittal direction, and 500 N vertical load plus a 10 Nm moment of right lateral direction were applied to the sacrum to simulate standing status, bending status, and flexion status, respectively. The maximum displacement value, the stress value, and the stress value of the fracture interface were compared among the three internal fixation techniques.

**Results:**

The results showed that all three internal fixation techniques effectively restored the biomechanical transmission of the injured pelvis. The stress on the implants in the TBP model was 167.47% and 53.41% higher than that in the ISS model and the MIAP model, respectively, and the stress shielding phenomenon of the TBP model was more obvious than in the other two models. Meanwhile, the stress between the fracture interfaces in the TBP fixation models was apparently higher than that in the other two models. However, the vertical displacement of the MIAP model was not significantly different from that in the ISS and TBP model; therefore, strong fixation could be obtained in all three models.

**Conclusion:**

Based on our results, we believe that the stability of Tile C pelvic fracture fixed with MIAP was similar to that of fractures fixed with ISS and TBP, but the stress shielding phenomenon and safety of implants in the TBP models were inferior to those in the MIAP and ISS fixation models. Meanwhile, MIAP and ISS fixation were more helpful to the healing processing than was TBP fixation, especially at the fracture interface of the second and third vertebral body levels.

## Introduction

The sacrum is the base of the spinal column as well as the keystone of the pelvic ring. Vertical fractures of the posterior pelvic ring have been associated with high rates of morbidity and haemorrhage^1^. Thus, the disrupted sacroiliac complex is an absolute indication for operative treatment. Various internal implants have been used to control these injuries, including iliosacral screws (ISS), tension band plates (TBP), and transiliac sacral bars. ISS has become a commonly used technology in the treatment of pelvic posterior ring disruption^2^. However, the ISS technique requires great surgical skill and continuous fluoroscopic guidance for appropriate screw insertion, and the risk of neurovascular injuries has been demonstrated to be higher than that for other techniques^3^, ^4^. A tension band plate placed in the posterior pelvic ring can provide sufficient stability for Tile C fractures^5^. However, the TBP technique has some disadvantages, including the necessity of pre‐bending the plate before fixation^6^; furthermore, it is associated with a high rate of skin infection and symptomatic hardware^7^. In addition, it is difficult to avoid repeated bending, which can reduce the plate strength and lead to damage of the threads of the screw holes in the locking compression plate (LCP)^8^.

To address the abovementioned limitations and to improve the reduction ability of posterior osteosynthesis, we introduced a novel minimally invasive adjustable plate (MIAP) for treating posterior pelvic ring disruption. The MIAP (Irene Medical Instrument Company, Tianjin, China) consists of two Z‐shaped brackets at each end of an adjustable connection bar. The Z‐shaped bracket is a single entity, consisting of three parts: an upper wing, a lower wing, and a web plate. The connection bar is comprised of a hexagonal tube and two custom‐made eye bolts. The hexagonal tube is threaded on the inside to allow a bolt to be screwed in from each end. The distance to which the eye bolts are screwed into the tube can be adjusted, and the length of the connection bar changes accordingly.

We used the 3D finite element (FE) analysis technique to imitate ISS, TBP, and MIAP fixation for a vertical sacral fracture (Tile C type) with high instability of the posterior pelvic ring under the following conditions: standing on both feet, during flexion, and during lateral bending. The study aimed to: (i) construct an FE model for posterior pelvic ring disruption that simulated human reality; (ii) analyze the influence of various internal implants on load transmission in posterior pelvic ring disruption; and (iii) observe the efficiency of the stabilization of these internal fixation techniques for treating posterior pelvic ring disruption and provide references for their clinical application.

## Materials and Methods

### *Geometric Definition of the Pelvis*

An anatomic pelvic model was constructed using CT (Philips Brilliance, Philips Healthcare, the Netherlands; slice thickness, 0.3 mm) data of a healthy woman (40 years old, 160 cm, 63 kg, no known history of bone disease or surgical interventions) to define the solid geometry of the pelvic bones and the upper one‐third of the femurs. The volunteer underwent a CT scan while both lower extremities were kept in a neutral position. The CT data were then imported into Mimics 10.0 medical image processing software (Materialise, Belgium) to construct the 3D surface mesh of the intact pelvis and input into Abaqus v 6.11 (Dassault Systemes Simulia, Providence, RI, USA) to perform the FE analysis. The ligaments could not be detected on CT, so they were constructed as 3D tension truss elements. The six main ligaments (sacrospinous ligament, sacrotuberous ligament, interosseous ligaments, sacroiliac anterior ligaments, sacroiliac dorsal ligaments, and arcuate pubic ligaments) were incorporated based on the significance of the pelvic ring ligaments on pelvic biomechanics determined in a previous study. This study was approved by the ethics committee of our institution.

### *Finite Element Model Construction*

The C3D4 was selected to mesh trabecular bone, and cortical bone was simulated on both the sacrum and the iliac bones by adding a 2.0‐mm thick shell element. Young’s modulus and Poisson’s ratio were taken to be 150 MPa and 0.2 for trabecular bone and 18 000 MPa and 0.3 for cortical bone, respectively. All material properties of bone were chosen in accordance with the results of a previous study^9^, ^10^. The material properties of the selected ligaments were assigned in accordance with the results of previous research and are listed in Table 1, 11, 12.

**Table 1:** The material properties of main ligaments in models

Material of ligament	Stiffness coefficient (N/mm)	Element number	Element type
Anterior sacroiliac ligament	700	20 × 2	Truss
Sacroiliac interosseous ligament	2800	20 × 2	Truss
Long posterior sacroiliac ligament	1000	20 × 2	Truss
Short posterior sacroiliac ligament	400	16 × 2	Truss
Sacrospinous ligament	1400	20 × 2	Truss
Sacrotuberous ligament	1500	15 × 2	Truss
Superior pubic ligaments	500	10	Truss
Arcuate pubic ligaments	500	10	Truss

In the anterior part of the pelvis, the symphysis pubis is covered with the superior and arcuate pubic ligaments. Meanwhile, its inter‐space is occupied by the interpubic disc, which is represented continuously and connects both sides of the ilium. The interpubic disc meshes into a tetrahedral element, and Young’s modulus and Poisson’s ratio were 5 MPa and 0.45, respectively^10^.

Finite element models of a disrupted posterior pelvic ring were constructed and cut into three parts through the left sacral foramina to develop a model of left vertical sacral fractures. Three kinds of internal fixation techniques were tested in this study (ISS, TBP, and MIAP) and were constructed in detail. In the ISS model, two sacroiliac screws with a diameter of 7.3 mm were fixed at S1 and S2 to simulate iliosacral screw fixation. In the TBP model, we developed an LCP and fixed it to the posterior superior iliac spines with six screws. In the MIAP model, two long screws were inserted into the ilium and two screws were inserted into the sacrum close to the sacroiliac joint to improve fixation stability.

Based on previous research^13^, six points on the surface of the pelvis were chosen as the measuring points. They were located along the pelvic biomechanical load‐transferring path from above downward: (i) the point on the first sacral vertebra near the sacroiliac joint; (ii) the point on the first sacral vertebra near the fracture gap; (iii) the point on the second sacral vertebra near the sacroiliac joint; (iv) the point on the second sacral vertebra near the fracture gap; (v) the midpoint of the iliopectineal line; and (vi) the dome region of the acetabulum.

In the FE models, the contact condition for the sacroiliac joint and hip joint was set as frictionless finite sliding contact pairs, the friction coefficient of the contacting pair was set at 0.015, and the initial penetration was set at 0.01 mm^14^. Meanwhile, a friction coefficient of 0.3 was applied between the interaction surfaces of fractures^15^.

When the pelvic FE models were simulated in the balanced standing phase, the constraints of the models were located on the ends of the proximal femurs, which were fixed in all directions to simulate standing on both feet. In each model, vertical loads of 500 N were added to the superior surface of the first sacral vertebral body to simulate upper body weight imposed on the sacrumin in the case of standing on both feet; the flexion state implemented a 500 N vertical load and a 10 Nm moment of forward sagittal direction; and the lateral bending state implemented a 500 N vertical load and a 10 Nm moment of right lateral direction.

### *Validation of the Intact and Injured Pelvic Finite Element Models*

The FE model of the intact pelvis was validated as follows. First, under the same experimental conditions as that of the two‐leg standing pelvis, the model predicted maximum vertical displacements (1.33–1.61 mm under 500 N vertical loads) that were coincident with the experiment‐measured maximum vertical displacements (0.973–1.550 mm under 500 N vertical loads) reported by Comstock *et al*.^16^. Second, to validate the developed FE model, we compared the strain values of specimen experiments with those of the pelvic FE model at each corresponding point by linear regression analysis. The loading and boundary conditions of the FE model were the same as those of the specimen experiments. The regression equation and correlation coefficient were obtained as follows: *y* = 1.025*x* − 0.958, *R* ^2^ = 0.971. The *x*‐axis represents the FE‐simulated equilibrium strains, and the *y*‐axis represents the strain values in the biomechanical experiment. The *R* ^2^ represents the correlation coefficient of the regression equation, which indicated that the FE analysis results had a strong correlation with the experimental results. Finally, the ISS model was validated as follows: when the femurs were not included, the calculations for FE models of ISS fixation showed that the vertical displacements were approximately 1.33 mm under 500 N vertical loads, which were close to the experimental results of approximately 1.69 mm under the same fixation models and load conditions measured by Zhao *et al*.^15^, who utilized two ISS to treat unilateral sacroiliac joint dislocation.

## Results

### *Efficacy of Three Internal Fixations Restoring the Biomechanical Transmission of the Injured Pelvis*

Under compression states, the minimum stress values of the six trial points were found at point 3, which were 0.9 MPa, 0.7 MPa, and 0.46 MPa in the ISS model, the TBP model, and the MIAP model, respectively; meanwhile, the maximum stress values of the six trial points were observed at point 6, which were 8.24 MPa, 8.8 MPa, and 8.19 MPa in the ISS model, the TBP model, and the MIAP model, respectively. Of the three models, the stress differences in the trial points, with the exception of point 2, were <10%. The stress values of point 2 in the MIAP model were diminished by 91.54% and 32.17% compared to those of the ISS model and the TBP model, respectively. The trends for stress distribution in the pelvis under lateral bending and flexion states were identical to those under compression.

**Figure 1:**
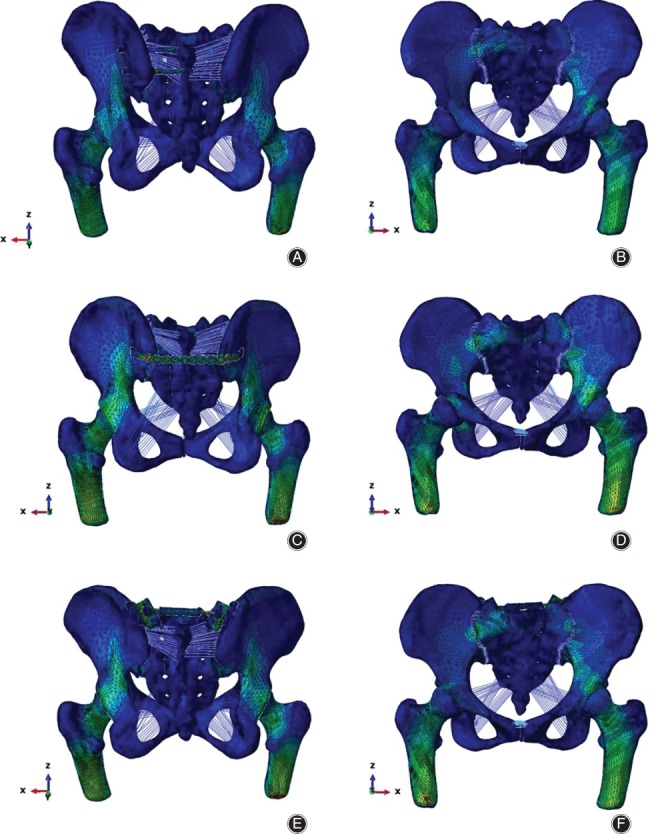
Stress distribution of the three models under compression states: the stress distribution is transmitted along the iliopectineal line in all three models, and the maximum stress value is less than the yield strength of bone. (A, B) In the ISS model, the maximum stress is located at the upper screw near the processus spinosus of the first sacral vertebra (anterior and posterior views). (C, D) In the TBP model, the maximum stress is located at the first screw near the right sacroiliac joint (anterior and posterior views). (E, F) In the MIAP model, the maximum stress is located at the upper screw near the sacroiliac joint (anterior and posterior). ISS, iliosacral screw; MIAP, minimally invasive adjustable plate; TBP, tension band plate.

The stress distribution of the pelvis was transmitted along the iliopectineal line in all three models under different motion states (Fig. 1).

### *Effect of Different Motion States on the Distribution of Stress*

The characteristics of stress distribution under different motion states were also compared in each model. In the ISS model, the minimum stress value of the trial points was located at point 2, which was 0.89 MPa, 0.85 MPa, and 0.85 MPa under compression, during lateral bending, and during flexion, respectively; furthermore, the maximum stress value of the trial points was located at point 5, which was 8.24 MPa, 8.14 MPa, and 8.14 MPa under compression, during lateral bending, and during flexion, respectively. The trends for stress distribution under different motion states in the TBP model and the MIAP model were identical to those in the ISS model.

### *Maximum Stress Values*

**Figure 2:**
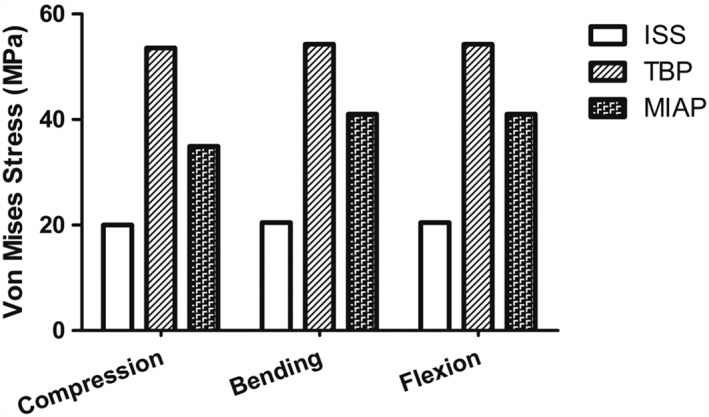
The maximum stress values of the implants in three different motion states: under compression, the maximum stress value of the implant in the TBP model is 167.47% and 53.41% higher than that in the ISS model and the MIAP model, respectively; under lateral bending states, the maximum stress value of the implant in the TBP model is 165.01% and 54.61% higher than that in the ISS model and the MIAP model, respectively; under flexion states, the maximum stress values of the implant in the TBP model is 165.14% and 54.61% higher than that in the ISS model and MIAP model, respectively. ISS, iliosacral screw; MIAP, minimally invasive adjustable plate; TBP, tension band plate.

Under all motion states for all three models, the stress concentrations were observed on the screws for all three implants; however, the maximum stress values of all the implants were less than their yield strengths (Fig. 2).

**Figure 3:**
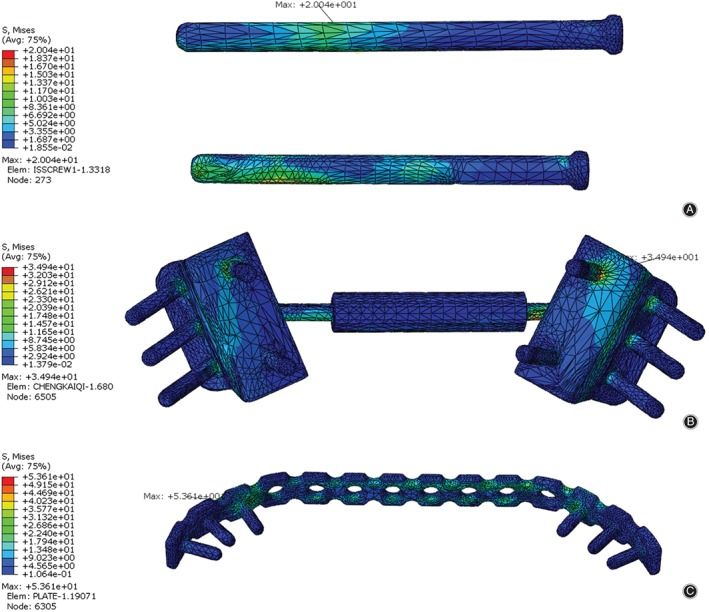
The stress distribution in the implants among the three models in a standing state. (A) In the ISS model, the maximum stress is located at the proximal screw near the spinous process of the first sacral vertebra. (B) In the MIAP model, the maximum stress is located at the proximal screw, which is located in the sacrum. (C) In the TBP model, the maximum stress is located at the first screw in right sacroiliac joint. ISS, iliosacral screw; MIAP, minimally invasive adjustable plate; TBP, tension band plate.

In simulating compression states, the maximum stress values of the implants were: 20.04 MPa, located at the proximal screw near the spinous process of the first sacral vertebra in the IS model; 53.6 MPa, located at the lateral screw which fixed in the right iliac bone in the TBP model; and 34.94 MPa, located at the proximal screw which fixed in the left sacral bone in the MIAP model, respectively, which, in the TBP model, was 167.47% and 53.41% higher than that in the ISS and MIAP models, respectively. The trends for maximum stress of the implants under the lateral bending and flexion states were identical to those under the compression states (Fig. 3).

### *Maximum Stress Values of Injured Sacrum*

Under compression, the maximum stress values of the injured sacrum were 10.09 MPa, 9.48 MPa, and 8.71 MPa in the ISS model, the TBP model, and the MIAP model, respectively; that in the MIAP model was diminished by 15.84% and 8.84% compared to those in the ISS model and TBP model, respectively. In addition, we found that in the three models, the maximum stress was located at the posteroinferior sacroiliac articular surface of the right sacrum. The trends for stress distribution of the injured sacrum under lateral bending states and flexion states were identical to that under compression.

### *Stress‐shielding Phenomenon*

**Figure 4:**
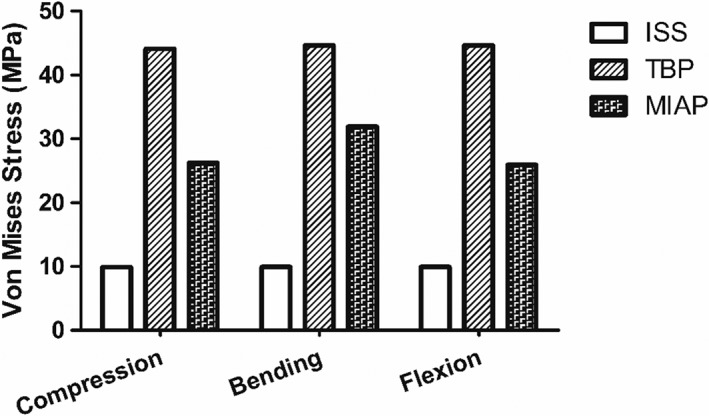
The comparison of the stress shielding among the three finite element models under different statuses. The stress shielding value of the TBP model was higher than that in the ISS and the MIAP models in all motion states. ISS, iliosacral screw; MIAP, minimally invasive adjustable plate; TBP, tension band plate.

We analyzed the effect of stress shielding originating from the implants to evaluate their biomechanical compatibility. The maximum stress difference between the implants and the injured sacrum indicates the degree of the stress‐shielding phenomenon. Under compression states, the maximum stress differences between the implants and the injured sacrum were 9.95 MPa, 44.12 MPa, and 26.23 MPa in the ISS, TBP, and MIAP models, respectively, and that in the TBP model was 343.42% and 68.2% higher than that in the ISS and MIAP models, respectively. The trends for the maximum stress difference between the implants and the injured sacrum under lateral bending states and flexion states were identical to those under compression (Fig. 4).

### *Difference in Vertical Displacement among the Three Internal Fixation Methods*

The maximal vertical displacement of the injured sacrum was 1.27 mm, 1.36 mm, and 1.24 mm in the ISS model, the TBP model, and the MIAP model under compression states, respectively. The degree of vertical displacement of the injured sacrum was ranked in sequence as TBP, ISS, and MIAP, and the TBP fixation type was only increased by 5.83% and 9.48% compared to that in the ISS and the MIAP models, respectively. The trends for maximum vertical displacement of the injured sacrum under lateral bending states and flexion states were identical to those under compression.

### *Maximum Stress of Vertebral Fracture Interface*

**Figure 5:**
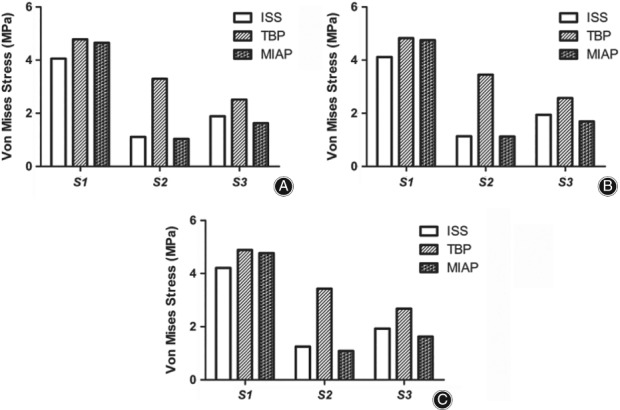
Stress at the fracture interface in different motion states: in all three motion states, the maximum stress between the fracture interface of the TBP model was higher than that in the others, especially in the second vertebral fracture interface. (A) Under compression status. (B) Under bending status. (C) Under flexion status.

Stress at the fracture interface is an important factor influencing the prognosis of fracture healing. With respect to the orientation of the maximum stress between the fracture interface, we found that the maximum stress was located at the upper part of each fractured medical vertebral body. Under compression states, the maximum stress of the first vertebral body fracture interface was greater in the TBP model than that in the ISS model and the MIAP model by 17.73% and 2.80%, respectively; on the second vertebral fracture interface, the maximum stress of the TBP model was 197.30% and 217.34% higher than that of the ISS and MIAP groups, respectively; and on the third vertebral fracture interface, the maximum stress of the TBP model was 33.33% and 54.6% higher than that of the ISS and MIAP groups, respectively. The trends of stress distribution between the fracture interface under lateral bending states and flexion states were identical to those under compression (Fig. 5).These results suggest that the TBP fixation models were, in general, inferior to the MIAP and ISS fixation models for sharing the stress of fracture interface, especially at the second and third vertebral body levels.

## Discussion

Posterior pelvic ring disruption remains a challenging problem for orthopaedic surgeons. The major surgical aim of posterior pelvic ring disruption is to promote the recovery of postoperative biomechanics of the posterior pelvic ring^17^. Therefore, the more that postoperative pelvic stress can approximate its natural stress, the better the clinical efficiency that can be achieved by an internal fixation system. Hao *et al*.^13^ developed an intact pelvic FE model to study the characteristics of stress distribution under a 500 N vertical load, and they found that the maximum stress values of the measuring points were observed at the point on the first sacral vertebra near the sacroiliac joint. However, in our study, the maximum stress value of the trial points was located at the dome region of the acetabulum under the same physiological loads, and the stress differences of this trial point in the three models was <10%. This phenomenon was probably due to the dome region of the acetabulum bearing the load transferred from the femur of the upper limb, which could cause stress concentration. At the same time, the dome region of the acetabulum was far away from the fixed position of the fracture so that there was no difference among the three models. We also found that at the point on the first sacral vertebra near the sacroiliac joint, the stress values were 2.24–4.27 MPa in the different models; however, Hao *et al*.^13^ found that the stress value in the same position was 15 MPa under a 550 N vertical load, which could indicate that the fixed pelvis will reduce stress concentration on the area near the sacroiliac joint. In contrast, in the ISS model, the stress values at the first sacral vertebra near the sacroiliac joint were significantly less than at the other two, which might be because the ISS were fixed in the first sacral vertebra body so as to reduce the stress transfer between SIJ at the level of the first vertebral body. Meanwhile, in many previous studies^12^, ^13^, researchers simulated an intact pelvic FE model and found the stress distribution along the iliopectineal line. In our study, according to a stress nephogram, the distribution of posterior pelvic stress was along the inner side of the pelvic ring, suggesting that the stress distribution of the pelvis was transmitted along the iliopectineal line in all three models under different motion states, similar to results for previous studies, and indicating that the transfer path of biomechanics had been restored by implants in our study.

The risk of implant breakage and loosening has increased while the maximum von Mises stress has increased. Fu *et al*.^18^ developed an FE model with bilateral vertical sacral fractures to compare the risk of breakage of two kinds of sacroiliac screws. They found that a fixed bilateral ilium and sacrum could decrease the risk of screw breakage to less than that for the fixed ilium. Sahin *et al*.^19^ compared the mechanical characteristics of the ISS and TBP fixation techniques. The average loads to failure for the ISS and TBP group were 1775 and 2230 N, respectively, and the average loads for 10 mm of displacement were 1033 and 2013 N. The data suggest that compared to the TBP model, the ISS model can reduce the risk of fixation failure. In our study, the maximum stress values of the implant in the TBP model were 165.14%–167.47% and 53.41%–54.61% higher than those in the ISS model and the MIAP model, respectively, under the same physiological loads. This finding showed that the implant stress concentration in the TBP model was stronger than that in the ISS and MIAP models, indicating that the risk of fatigue injury and screw loosening for the ISS system and the MIAP system were less than those for the TBP system. We speculated that the MIAP and two ISS not only fixed the sacrum but also fixed both sides of the ilium, which functioned as a suspension bridge structure similar to the sacroiliac complex, which could make the stress on the implant more dispersed. Furthermore, the maximum von Mises stress of implants is one of the important indices used to represent implant safety. The yield stress of titanium alloy is 1050 MPa^20^. In our study, the maximum stress values of the implants were 20.04–54.3 MPa under a 500 N vertical load when different internal fixations were simulated under all motion states, showing that even the maximum stress value of implants was less than the yield stress of the material. This finding suggests that screw and plate breakage due to the high peak value of implants in the three internal fixations could not have been anticipated.

A higher peak stress value on the bone, especially the bone around screws, may lead to implant loosening and secondary fractures. Böhme *et al*.^21^ simulated a patient‐specific FE model based on an actual case to speculate on the clinical rehabilitation course. According to the radiologic examination of the clinical process, implant loosening as well as newly occurring fractures were shown to coincide with regions with the highest stress levels. To probe the yield stress of the pelvis, Li *et al*.^22^ developed an FE model to study the biomechanical response of the pelvis during lateral impact, and they found that the yield strength of the sacrum was 150–158 MPa; otherwise, secondary fractures could occur when the peak stress value of the bone was greater than the upper limit. In our study, the maximum stress value of the pelvic bone with the three internal fixation methods was much lower than the aforementioned yield stress, suggesting that implant loosening and secondary fracture due to the peak value of the sacrum in the three internal fixations could not have been anticipated.

According to the principle of stress shielding, the smaller the stress difference between the pelvis and the internal implants becomes, the better the biomechanical compatibility that can be achieved by an internal implants system^23^. Chen *et al*.^24^ developed two groups of FE models for Denis I–III type vertical sacral fractures treated with either a percutaneous metallic plate or a percutaneous screw to explore the biomechanical compatibility, and they found that the biomechanical compatibility of percutaneous plate fixation models was better than percutaneous screw fixation models for the treatment of Denis III type sacral factures. In our study, the maximum stress difference between the implants and pelvis in the TBP model was 343.42%–349.09% and 68.2%–72.19% higher than that in the ISS and the MIAP model, respectively, under the same physiological loads. This finding indicated that the stress‐shielding phenomenon of the TBP model was more obvious than in the other two models, which may be because the TBP was only fixed in the bilateral posterior superior iliac spines, which could lead to the compressive stress concentrated on the implants, making the stress‐shielding phenomenon of the TBP model more obvious there than in the other two models.

In many previous studies, the maximum stress of the sacrum in the intact pelvis FE model has been observed. Hao *et al*.^13^developed an intact pelvic FE model. The maximum stress value of the intact sacrum was 15 MPa under a 550 N vertical load, which was located at the sacral joint. Shi *et al*.^14^ established an intact pelvic FE model with synovial conditions in the sacroiliac joint, and they found that the maximum stress of the sacrum was 16.5 MPa under a 500 N vertical load. However, in our study, the maximum stress values of the injured sacrum were 8.71–10.5 MPa under a 500 N vertical load when different internal fixations under all motion states were simulated. Our maximum stress was less than that reported in previous articles, and we believe that the gap between ours and the previous studies was mainly due to the stress sharing of implants from the posterior pelvic ring. Furthermore, we found that the maximum stress values of the injured sacrum in the MIAP model were diminished compared to those in the ISS model and TBP model. We speculated that as the MIAP fixed in the bilateral posterior superior iliac spines and the bilateral sacral cortex, compared with the TBP fixed bilateral iliac and ISS fixed unilateral iliac and sacral, therefore, the MIAP can distribute the stress to both sides of the ilium and sacrum, which could avoid the stress concentration of the sacrum.

In the injured pelvis model, the maximum displacement of sacral bone was the summation of the combined displacement of the sacroiliac joint and fracture interface. Osterhoff *et al*.^25^ investigated the biomechanical stability of implant fixation in an open book injury. Under a 200 N vertical load, the displacement in the sacroiliac joint area had a mean 0.156 mm. We believe that if the load were to increase, the displacement of the joint would be far greater than 0.156 mm. Furthermore, Shi *et al*.^14^ studied the effect of contact condition on the displacement of the intact pelvis, and found that the maximum displacement of the sacral bone was 1.3 mm under a 500 N vertical load when the sacroiliac joint was set as the synovial condition. Meanwhile, they found that the maximum displacement of the iliac bone was 0.45 mm, so the movement of the sacroiliac joint was approximately 0.85mm. In our study, the maximum displacement of the sacral bone was 1.16–1.36 mm under a 500 N vertical load, which means that the displacement of the sacroiliac joint and interfragmentary is equal to 1.16–1.36 mm. The results showed that the displacement of the fracture interface was less than 0.31–0.51 mm. A nominal failure vertical fracture interface displacement of 2 mm was judged to be clinically relevant^26^; thus, we believe that the three kinds of internal fixation can achieve stable fixation. However, in clinical cases, the smaller the postoperative displacement of the pelvic ring becomes, the better the biomechanical stability that will be obtained through internal fixation. The long‐term functional outcome seems to be improved if reduction with <1 cm displacement of the posterior pelvic ring is obtained. The degree of vertical displacement of the injured sacrum was different in the three models and ranked sequentially as TBP, ISS, and MIAP; thus, MIAP fixation produced minimal vertical displacement of the sacrum. However, the vertical displacement of the sacrum was not significantly different among the three groups. In our view, these three kinds of internal fixation resulted in the same effects on the biomechanical stability of the posterior pelvic ring.

The presence of a small amount of stress in the fracture interface can promote healing of the fracture. According to a biomechanical experiment, Claes *et al*.^27^ found that in the healing process of a fracture, intramembranous bone occurred only in low‐strain and low‐stress statuses, usually at 0.25–1 MPa, which occurs in a mechanical environment that could promote osteoblast proliferation and activation. However, excessive stress had a negative effect on the proliferation and differentiation of osteoblasts. The sacral fracture is a kind of cancellous bone fracture; thus, the stress concentration on the fracture interface is undoubtedly an adverse condition for the healing process. In our study, the maximum stress of the second and third vertebral fracture interface in the TBP model was remarkably higher than that in the ISS and MIAP models under the same physiological loads. We speculated that in TBP fixation, compression stress on both iliac bones, which was exerted by the plate, was necessary for stabilizing the fracture to result in an increased peak value of the stress on the fracture interface. Furthermore, TBP was positioned at the level of the second vertebral body, and the stress peak value of the second vertebral fracture interface indicated a relationship between the peak value and TBP fixation. Thus, MIAP and ISS fixation were more helpful to the healing processing than was TBP fixation, especially at the fracture interface of the second and third vertebral body levels.

### *Limitations*

The present FE models still contain certain approximations and limitations. First, in our FE model, the effects of pelvic muscles and fascia on pelvic stability were not taken into consideration, nor was the difference in pelvic bony density that may have caused the difference in pelvic biomechanics *in vivo.* However, all surgical FE models of TBP, MIAP, and ISS fixations were simulated in the same experimental conditions *in vitro* with the same bony material properties, which could reliably distinguish between the mechanical differences of these three internal fixations. Second, this study was based on an anatomic reduction achieved with surgery. If the reduction were non‐anatomic, the results of this study would no longer be valid. Third, bone mineral density was not considered in this model. One would expect an osteoporotic model to show greater displacement.

### *Conclusions*

In conclusion, the biomechanical stability was not significantly different for the three types of internal fixation; however, the risks of fatigue injury and screw loosening and the stress‐shielding phenomenon of the ISS system and the MIAP system were lower than that for the TBP system. Meanwhile, MIAP and ISS fixation were more helpful to the healing processing than was TBP fixation, especially at the fracture interface of the second and third vertebral body level. In clinical practice, the MIAP technique is easy to perform and requires less radiation exposure than other techniques; it is technically safe and saves time, and the biomechanical stability of MIAP was similar to that for percutaneous IS screw fixation and the TBP technique, suggesting that the MIAP is a good option for treating posterior pelvic ring fractures and sacroiliac joint dislocations.
